# Predicting Depressive Symptom Severity Through Individuals’ Nearby Bluetooth Device Count Data Collected by Mobile Phones: Preliminary Longitudinal Study

**DOI:** 10.2196/29840

**Published:** 2021-07-30

**Authors:** Yuezhou Zhang, Amos A Folarin, Shaoxiong Sun, Nicholas Cummins, Yatharth Ranjan, Zulqarnain Rashid, Pauline Conde, Callum Stewart, Petroula Laiou, Faith Matcham, Carolin Oetzmann, Femke Lamers, Sara Siddi, Sara Simblett, Aki Rintala, David C Mohr, Inez Myin-Germeys, Til Wykes, Josep Maria Haro, Brenda W J H Penninx, Vaibhav A Narayan, Peter Annas, Matthew Hotopf, Richard J B Dobson, RADAR-CNS Consortium

**Affiliations:** 1 Department of Biostatistics & Health Informatics Institute of Psychiatry, Psychology and Neuroscience King's College London London United Kingdom; 2 Institute of Health Informatics University College London London United Kingdom; 3 NIHR Biomedical Research Centre at South London and Maudsley NHS Foundation Trust and King’s College London London United Kingdom; 4 Health Data Research UK London University College London London United Kingdom; 5 NIHR Biomedical Research Centre at University College London Hospitals NHS Foundation Trust London United Kingdom; 6 Department of Psychological Medicine Institute of Psychiatry, Psychology and Neuroscience King's College London London United Kingdom; 7 Department of Psychiatry Amsterdam Public Health Research Institute and Amsterdam Neuroscience Amsterdam University Medical Centre, Vrije Universiteit and GGZ InGeest Amsterdam Netherlands; 8 Teaching Research and Innovation Unit Parc Sanitari Sant Joan de Déu Fundació Sant Joan de Déu Barcelona Spain; 9 Centro de Investigación Biomédica en Red de Salud Mental Madrid Spain; 10 Faculty of Medicine and Health Sciences Universitat de Barcelona Barcelona Spain; 11 Department of Psychology Institute of Psychiatry, Psychology and Neuroscience King's College London London United Kingdom; 12 Center for Contextual Psychiatry Department of Neurosciences Katholieke Universiteit Leuven Leuven Belgium; 13 Faculty of Social Services and Health Care LAB University of Applied Sciences Lahti Finland; 14 Center for Behavioral Intervention Technologies Department of Preventive Medicine Northwestern University Evanston, IL United States; 15 Janssen Research and Development LLC Titusville, NJ United States; 16 H Lundbeck A/S Copenhagen Denmark

**Keywords:** mental health, depression, digital biomarkers, digital phenotyping, digital health, Bluetooth, hierarchical Bayesian model, mobile health, mHealth, monitoring

## Abstract

**Background:**

Research in mental health has found associations between depression and individuals’ behaviors and statuses, such as social connections and interactions, working status, mobility, and social isolation and loneliness. These behaviors and statuses can be approximated by the nearby Bluetooth device count (NBDC) detected by Bluetooth sensors in mobile phones.

**Objective:**

This study aimed to explore the value of the NBDC data in predicting depressive symptom severity as measured via the 8-item Patient Health Questionnaire (PHQ-8).

**Methods:**

The data used in this paper included 2886 biweekly PHQ-8 records collected from 316 participants recruited from three study sites in the Netherlands, Spain, and the United Kingdom as part of the EU Remote Assessment of Disease and Relapse-Central Nervous System (RADAR-CNS) study. From the NBDC data 2 weeks prior to each PHQ-8 score, we extracted 49 Bluetooth features, including statistical features and nonlinear features for measuring the periodicity and regularity of individuals’ life rhythms. Linear mixed-effect models were used to explore associations between Bluetooth features and the PHQ-8 score. We then applied hierarchical Bayesian linear regression models to predict the PHQ-8 score from the extracted Bluetooth features.

**Results:**

A number of significant associations were found between Bluetooth features and depressive symptom severity. Generally speaking, along with depressive symptom worsening, one or more of the following changes were found in the preceding 2 weeks of the NBDC data: (1) the amount decreased, (2) the variance decreased, (3) the periodicity (especially the circadian rhythm) decreased, and (4) the NBDC sequence became more irregular. Compared with commonly used machine learning models, the proposed hierarchical Bayesian linear regression model achieved the best prediction metrics (*R^2^*=0.526) and a root mean squared error (RMSE) of 3.891. Bluetooth features can explain an extra 18.8% of the variance in the PHQ-8 score relative to the baseline model without Bluetooth features (*R^2^*=0.338, RMSE=4.547).

**Conclusions:**

Our statistical results indicate that the NBDC data have the potential to reflect changes in individuals’ behaviors and statuses concurrent with the changes in the depressive state. The prediction results demonstrate that the NBDC data have a significant value in predicting depressive symptom severity. These findings may have utility for the mental health monitoring practice in real-world settings.

## Introduction

Existing studies have demonstrated that depression is significantly associated with individuals’ behaviors and statuses, such as social connections and interactions, working status, mobility, and social isolation and loneliness [1-4]. For example, individuals reporting fewer social network connections or less social support tend to have higher depressive symptomatology [1]. As the depressive mood and medical comorbidity can make people unable to work, the unemployment rate in depression is high [2]. Reduced mobility and physical activity are associated with depressive symptoms [3]. Loneliness is a specific risk factor for depression, and a significant proportion of suicides have a history of social isolation [1,4]. Although these findings have been replicated in different populations, these studies relied on participant self-report, which is susceptible to recall bias and typically does not capture dynamic information [5].

Mobile phone technology provides an unobtrusive, continuous, and cost-efficient means to capture individuals’ daily behaviors and statuses using a number of embedded sensors, such as accelerometers, GPS sensors, and Bluetooth sensors [6]. The embedded Bluetooth sensor can be used to record individuals’ local proximity information, such as the nearby Bluetooth device count (NBDC) that includes the Bluetooth signal of other phone users [7]. The continuously recorded NBDC data represents a mixed signal that has been used to estimate individuals’ behaviors and statuses, including face-to-face social interactions [8-10], working status [11], mobility [12], and isolation and loneliness [13,14]. Therefore, the NBDC data have the potential to reflect changes in people’s behaviors and statuses during the depressive state.

There have been a few studies exploring the relationship between the NBDC data and depression directly. Wang et al found a negative association (*r*=−0.362, *P*=.03) between the NBDC and self-reported depressive symptoms on the StudentLife data set, which contained mobile phone data from 48 students across a 10-week term at Dartmouth College [15]. Boonstra et al illustrated the feasibility of collecting nearby Bluetooth device information for the depression recognition task, but they did not provide further findings [5].

Several recent studies have investigated the relationships between Bluetooth proximity data and mental health [16-18]. Moturu et al found that individuals with lower sociability (estimated by the NBDC) tend to report lower mood more often [16]. Bogomolov et al established machine learning models to recognize happiness and stress with features of Bluetooth records, calls, and text messages, which obtained accuracy rates of 80.81% and 72.28%, respectively [17,18]. The above three studies were all performed on the “Friends and Family” data set, including 8 weeks of mobile phone data from 117 participants living in a major US university’s married graduate student residency.

Previous studies [15-18] have been performed on relatively small (approximately 100 participants) homogeneous (eg, university students) cohorts of participants over relatively short periods (8-10 weeks), which may limit their generalizability. Besides, Bluetooth features used in these studies [15-18] have been limited to basic statistical features (eg, sum, mean, and standard deviation), which are unable to characterize some nonlinear aspects (such as complexity, regularity, and periodicity) of the Bluetooth data. These nonlinear characteristics can reflect individuals’ life rhythms, such as circadian and social rhythms, which are affected by depressive symptoms [19]. Therefore, the associations between the NBDC data and depression are yet to be fully explored.

In this paper, we aimed to explore the value of the NBDC data in predicting self-reported depressive symptom severity in a relatively large cohort of individuals with a history of recurrent major depressive disorder. Our first objective was to explore the associations between statistical Bluetooth features and depressive symptom severity. Our second objective was to extract nonlinear features for quantifying complexity, regularity, and periodicity from the NBDC data and test their associations with depression. The third objective was to leverage appropriate machine learning models to predict the severity of depressive symptoms using extracted Bluetooth features.

## Methods

### Data Set

#### Study Participants and Settings

The data used in this study were collected from a major EU Innovative Medicines Initiative (IMI) research program Remote Assessment of Disease and Relapse-Central Nervous System (RADAR-CNS) [20]. The project aimed to investigate the use of remote measurement technologies (RMTs) to monitor people with depression, epilepsy, and multiple sclerosis in real-world settings. The study protocol for the depression component (Remote Assessment of Disease and Relapse-Major Depressive Disorder; RADAR-MDD) has been described in detail by Matcham et al [21]. The RADAR-MDD project aimed to recruit 600 participants with a recent history of depression from three study sites in Spain (Centro de Investigación Biomédican en Red [CIBER], Barcelona), the Netherlands (Vrije Universiteit Medisch Centrum [VUmc], Amsterdam]), and the United Kingdom (King’s College London [KCL]). Recruitment procedures varied slightly across sites with eligible participants identified through existing research infrastructures (in KCL and VUmc) where consent to be contacted for research purposes exists; advertisements in general practices, psychologist practices, and newspapers; Hersenonderzoek.nl [22], a Dutch online registry (VUmc); and mental health services (in KCL and CIBER) [21].

Participants were asked to install passive and active remote monitoring technology (pRMT and aRMT, respectively) apps and use an activity tracker for up to 2 years of follow-up. Many categories of passive and active data were collected and uploaded to an open-source platform, RADAR-base [23].

As the purpose of this paper was to explore the value of the NBDC data in predicting self-reported depressive symptom severity, we focused on the NBDC data, 8-item Patient Health Questionnaire (PHQ-8) data [24], and baseline demographics. However, according to our previous research, the COVID-19 pandemic and related lockdown policies greatly impacted the behaviors (particularly mobility, social interactions, and working environment [working from home]) of European people [25]. To exclude the impact of the COVID-19 pandemic, we performed a preliminary analysis with the data before February 2020.

#### PHQ-8 Data

The variability of each participant’s depressive symptom severity was measured via the PHQ-8, conducted by mobile phones every 2 weeks. The PHQ-8 score ranges from 0 to 24 (increasing severity) [24]. According to the PHQ-8 score, the severity of depression can usually be divided into the following five levels: asymptomatic (PHQ-8 <5), mild (5 ≤ PHQ-8 < 10), moderate (10 ≤ PHQ-8 < 15), moderately severe (15 ≤ PHQ-8 < 20), and severe (PHQ-8 ≥20) [24].

#### NBDC Data

The RADAR-base pRMT app scanned other Bluetooth devices in the participant’s physical proximity once every hour. To avoid privacy leaks from participants and passers, the Media Access Control (MAC) address and types of Bluetooth devices were not recorded in this study. The NBDC was uploaded to the RADAR-base platform for further analyses.

**Figure 1:**
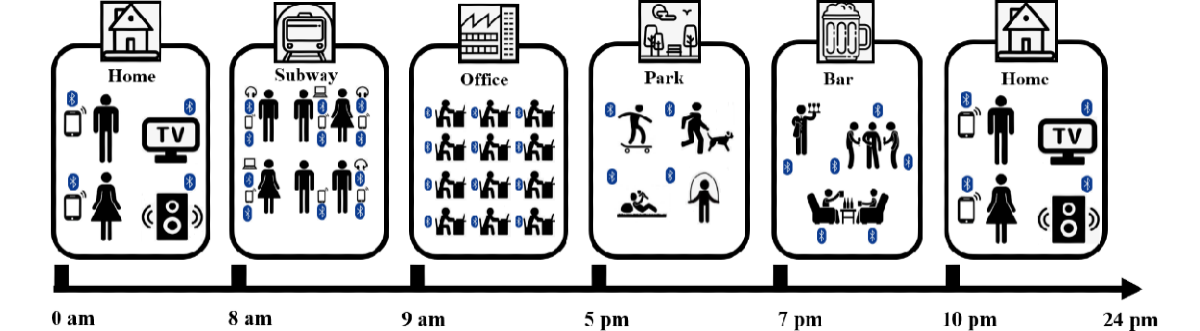
A schematic diagram showing an individual’s nearby Bluetooth devices count (NBDC) in different scenarios in daily activities and life.

Figure 1 is a schematic diagram showing an individual’s NBDC in different scenarios in daily activities and life. At home, the NBDC is related to the number of family members and Bluetooth devices in the house, reflecting the participant’s connections with family (whether living alone) and the number of other Bluetooth devices. In public transportation (such as the train, subway, and bus), the NBDC is affected by the number of surrounding passengers’ Bluetooth devices, reflecting the participant’s social connections with strangers. Studies have shown that whether feeling comfortable in the presence of strangers is related to the intensity of social connections [26]. In the company, the NBDC can reflect the participant’s social connections and interactions with co-workers. After work, the NBDC can reflect whether the participant joins other social activities, such as going to the park or bar. Therefore, the NBDC data contain information about participants’ social connections and interactions with family, friends, co-workers, and strangers, and the data can also reflect participants’ time at home, mobility, social isolation, and working status, as well as the number of other Bluetooth devices in the house and working environment.

**Figure 2:**
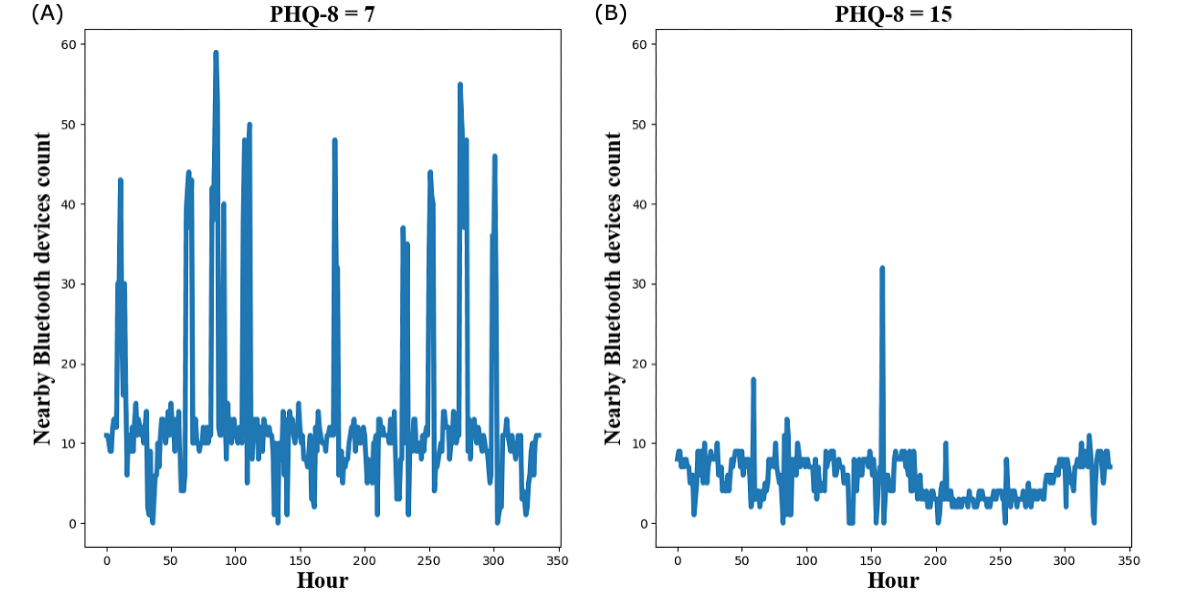
An example of two 14-day nearby Bluetooth devices count (NBDC) sequences from the same participant at the mild depression level (A) and moderately severe level (B). PHQ-8: 8-item Patient Health Questionnaire.

Figure 2 shows an example of two NBDC sequences collected over 14 days (336 hours) before two PHQ-8 records from one participant at two different depression severity levels (mild vs moderately severe).

#### Demographics

Participants’ demographics were recorded during the enrollment session. According to previous studies [27,28], baseline age, gender, and education level were considered as covariates in our analyses. Due to the different educational systems in the three countries in our data set, we used the number of years in education to represent education level.

#### Data Inclusion Criteria and Data Preprocessing

For each PHQ-8 record, we considered a “PHQ-8 interval” of 14 days before the day when the participant fills in the PHQ-8 questionnaire, as the PHQ-8 score is used to represent the depressive symptom severity of the participant for the past 2 weeks. To reduce the impact of the COVID-19 pandemic and missing data on our analysis, we specified the following two data inclusion criteria:

1. As mentioned in the data set section, to exclude the impact of the COVID-19 pandemic, we restricted our analysis to PHQ-8 records prior to February 2020.
2. Saeb et al [29] and Farhan et al [30] used 50% as each day’s completeness threshold for passive data. In our data set, 89.62% of days have 50% (12 hours) or more of the NBDC data. We considered one day as a “valid day” if it contained at least 12 hours of the NBDC data. Then, we empirically selected PHQ-8 intervals with at least 10 valid days as valid PHQ-8 intervals to retain the majority (81.78%) of PHQ-8 intervals.

For the NBDC sequence in each selected PHQ-8 interval, we used linear interpolation to impute the missing hours in all valid days and discarded the NBDC data that did not belong to a valid day. The “NBDC sequence” in the rest of this paper refers to the preprocessed NBDC data in the 14-day PHQ-8 interval.

### Feature Extraction

According to past Bluetooth-related research [15-18] and research on nonlinear features of signal processing [31,32], we extracted 49 Bluetooth features from the NBDC sequence in the PHQ-8 interval in the following three categories: second-order statistics, multiscale entropy (MSE), and frequency domain (FD). Table 1 summarizes all Bluetooth features extracted in this paper.

**Table 1:** Summary of 49 Bluetooth features used in this paper and their short descriptions.

Category	Abbreviation	Description	Number of features (N=49)
Statistical features	[Second-order feature]_[Daily feature], eg, Max_Mean	Second-order features (max, min, mean, and standard deviation) calculated in the PHQ-8^a^ interval based on daily statistical Bluetooth features (max, min, mean, and standard deviation).	16
Multiscale entropy (MSE)	MSE_1, MSE_2, …, MSE_24	Multiscale entropy of the NBDC^b^ sequences from scale 1 to scale 24.	24
Frequency domain^c^	LF_sum, MF_sum, HF_sum	The sums of spectrum power in LF, MF, and HF.	3
Frequency domain	LF_pct, MF_pct, HF_pct	The percentages of spectrum power in LF, MF, and HF to the total spectrum power.	3
Frequency domain	LF_se, MF_se, HF_se	Spectral entropy in LF, MF, and HF.	3

^a^PHQ-8: 8-item Patient Health Questionnaire.

^b^NBDC: nearby Bluetooth device count.

^c^LF: low frequency (0-0.75 cycles/day); MF: middle frequency (0.75-1.25 cycles/day); HF: high frequency (>1.25 cycles/day).

#### Second-Order Statistical Features

We first calculated four daily features (max, min, mean, and standard deviation) of daily NBDC data from all valid days in the PHQ-8 interval. For each daily feature, we calculated four second-order features (max, min, mean, and standard deviation) to reflect the amount and variance of the NBDC in the PHQ-8 interval. These features were denoted in the following format: [Second-order feature]_[Daily feature]. For example, the average value of the daily maximum number of the NBDC in the PHQ-8 interval was denoted as *Mean_Max*. A total of 16 second-order statistical features were extracted.

#### Nonlinear Bluetooth Features

The second-order statistical features can only reflect the amount (max, min, and mean) and variance (standard deviation) of the NBDC data. To exploit more information embedded in the NBDC data, we proposed MSE and FD features to measure the nonlinear characteristics, such as regularity, complexity, and periodicity, of the NBDC sequence.

##### Multiscale Entropy Features

MSE analysis has been used to provide insights into the complexity and periodicity of signals over a range of timescales since the method was proposed by Costa et at [31]. It has been widely used in the field of signal analysis, such as heart rate variability analysis [33], electroencephalogram analysis [34], and gait dynamics analysis [35]. Compared with other entropy techniques (eg, sample entropy and approximate entropy), the advantage of MSE analysis is that the assessments of complexity at shorter and longer timescales can be analyzed separately [36]. The MSE at short timescales reflects the complexity of the sequence. The larger the MSE at short timescales, the more chaotic and irregular the signal. The MSE at relatively long timescales assesses fluctuations occurring at a certain period, reflecting the periodicity of the signal.

**Figure 3:**
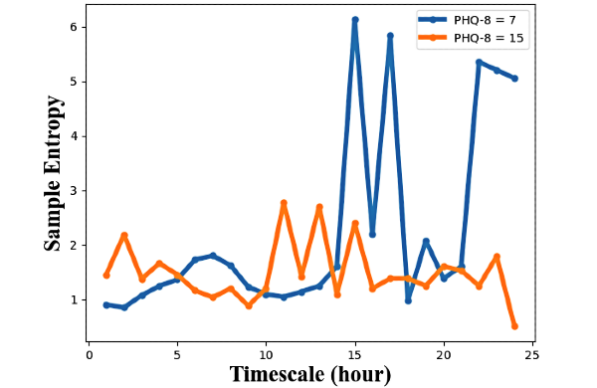
An example of multiscale entropy (scale 1-24) of two 14-day nearby Bluetooth device count (NBDC) sequences at the mild depression level (blue) and the moderately severe level (orange) from the same participant as in Figure 2. PHQ-8: 8-item Patient Health Questionnaire.

To explore the complexity and periodicity of the NBDC sequence on different timescales (from 1 hour to 24 hours), we calculated MSE features of the NBDC sequences from scale 1 to scale 24, denoted as *MSE_1*, *MSE_2*, *…*, *MSE_24*. Figure 3 shows an example of MSE features calculated on two NBDC sequences at different depression severity levels from the same participant shown in Figure 2. In this example, the NBDC sequence at the mild depression level (PHQ-8=7) has lower MSE at relatively short timescales (scale 1-3) and higher MSE at relatively long timescales than the sequence at the moderately severe depression level (PHQ-8=15). This indicated that this participant’s NBDC sequence at the mild depression level was more regular and periodic than the NBDC sequence at the moderately severe depression level.

##### FD Features

FD analysis has been widely used in the signal processing field, especially for signals with periodic characteristics [32]. People’s behaviors follow a quasiperiodic routine, such as sleeping at night, working on weekdays, and gathering with friends on weekends [19,37]. We therefore leveraged FD analysis to explore the periodic patterns in the NBDC data. Fast Fourier transformation (FFT) was performed to transform the NBDC sequence from the time domain to the FD. We set the sample rate to 24 hours, and then, the spectrum generated by FFT had the frequency axis scaled to reflect cycles per day.

**Figure 4:**
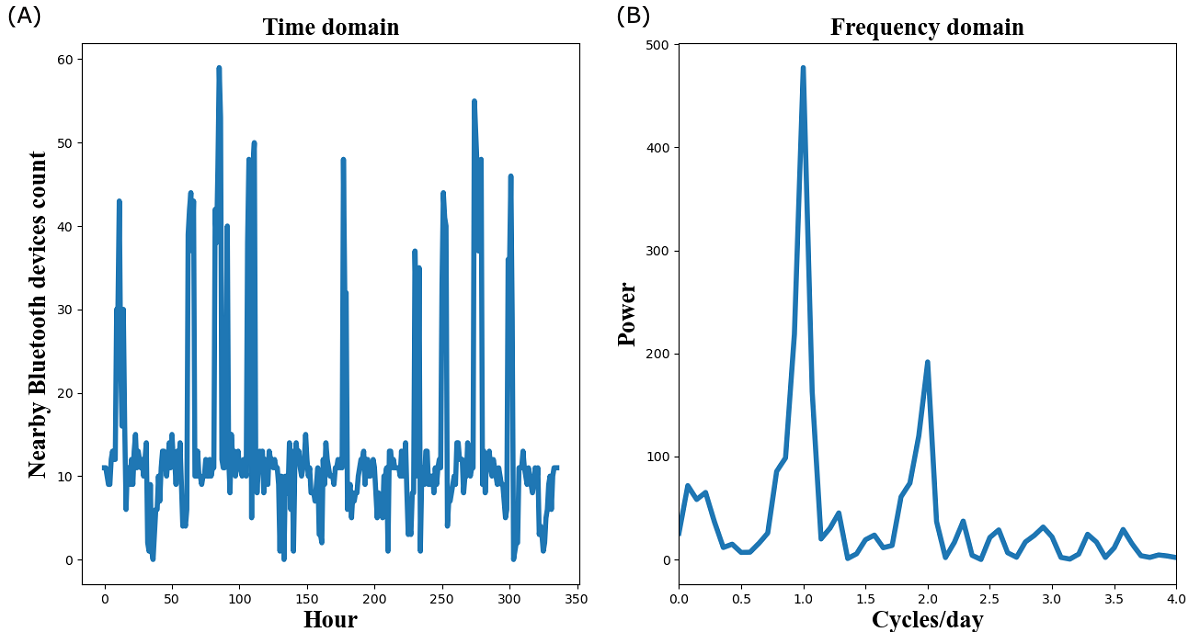
An example of a 14-day nearby Bluetooth devices count (NBDC) sequence in the time domain (A) and its spectrum in the frequency domain (B).

Figure 4 is an example of a NBDC sequence in the time domain and its spectrum in the FD. According to the spectrum’s definition, spectrum power around 1 cycle per day reflects the participant’s circadian rhythm (approximately 24-hour rhythm) [19]. To explore the periodic rhythms of different period lengths, we empirically defined the following three frequency intervals: low frequency (LF) (0-0.75 cycles/day), middle frequency (MF) (0.75-1.25 cycles/day), and high frequency (HF) (>1.25 cycles/day). The power in MF represents the circadian rhythm. Similarly, the power in LF represents the long-term (>1 day) rhythm, while the power in HF represents the short-term (<1 day) rhythm.

The sums of spectrum power in these three frequency intervals were calculated and denoted as *LF_sum*, *MF_sum*, and *HF_sum*, respectively. The percentages of spectrum powers in these three frequency intervals to the total spectrum power were extracted and denoted as *LF_pct*, *MF_pct*, and *HF_pct*, respectively. To estimate the complexity and regularity of the spectrum, we calculated spectral entropy (SE) [38] in these three intervals, denoted as *LF_se*, *MF_se*, and *HF_se*, respectively.

### Statistical Methods

The linear mixed-effect model contains both fixed and random effects, allowing for both within-participant and between-participants variations over repeated measurements [39]. Therefore, we used linear mixed-effect models in our statistical analyses.

#### Pairwise Association Analyses

To explore the association between each Bluetooth feature and depression severity, a series of pairwise linear mixed-effect models with random participant intercepts were performed to regress the PHQ-8 score with each of the Bluetooth features. All mixed-effect models, baseline age, gender, and years in education were considered as covariates. The z-test was used to evaluate the statistical significance of the coefficient of each model. The Benjamini-Hochberg method [40] was used for correction of multiple comparisons, and the significant level for the adjusted *P* value was set to .05. All linear mixed-effect models were implemented by using the R package “lmerTest,” and the Benjamini-Hochberg method was performed by using the command “p.adjust” in R software (R Foundation for Statistical Computing).

#### Likelihood Ratio Test

One objective of this paper was to assess what value these Bluetooth features provide beyond other information that might be readily available, such as baseline demographics. The likelihood ratio test is a statistical test of goodness of fit between two nested models [41]. If the model with more parameters fits the data significantly better, it indicates that additional parameters provide more information and improve the model’s fitness [41]. Therefore, we built three nested linear mixed-effect models with random participant intercepts (model A, model B, and model C). The predictors of model A were only demographics. The predictors of model B were demographics and 16 second-order statistical features. The predictors of model C were demographics and all 49 Bluetooth features. The likelihood ratio tests were performed to test whether these Bluetooth features have a significant value in fitting the PHQ-8 score regression model.

### Prediction Models

Another objective of this paper was to examine whether it is possible to predict participants’ depressive symptom severity using Bluetooth features combined with some known information (demographics and previous PHQ-8 scores). A subset of PHQ-8 intervals was selected for the prediction task based on the following two additional criteria:

1. To ensure that each participant had sufficient PHQ-8 intervals for the time-series cross-validation (described in the following model evaluation section), the number of valid PHQ-8 intervals for each participant should be at least 3.
2. To test whether the model can predict variability of depression severity, the difference of one participant’s PHQ-8 scores should be more than or equal to 5 (clinically meaningful change) [42].

#### Hierarchical Bayesian Linear Regression Model

The hierarchical Bayesian approach is an intermediate method compared to the completely pooled model and individualized model, capturing the whole population’s characteristics while allowing individual differences [43]. We leveraged the hierarchical Bayesian linear regression model to predict participants’ PHQ-8 scores using Bluetooth features, demographics (age, gender, and years in education), and the last observed PHQ-8 score. In this study, we implemented the hierarchical Bayesian linear regression using the “PyMC3” package [44] in Python. To compare the results with other commonly used machine learning models, we also implemented the LASSO regression model [45] and XGBoost regression model [46] using the Scikit-learn machine learning library [47] in Python. As depressive mood has a strong autocorrelation [48], we considered a baseline hierarchical Bayesian linear regression model with the last observed PHQ-8 score and demographics as predictors.

#### Model Evaluation

**Figure 5:**
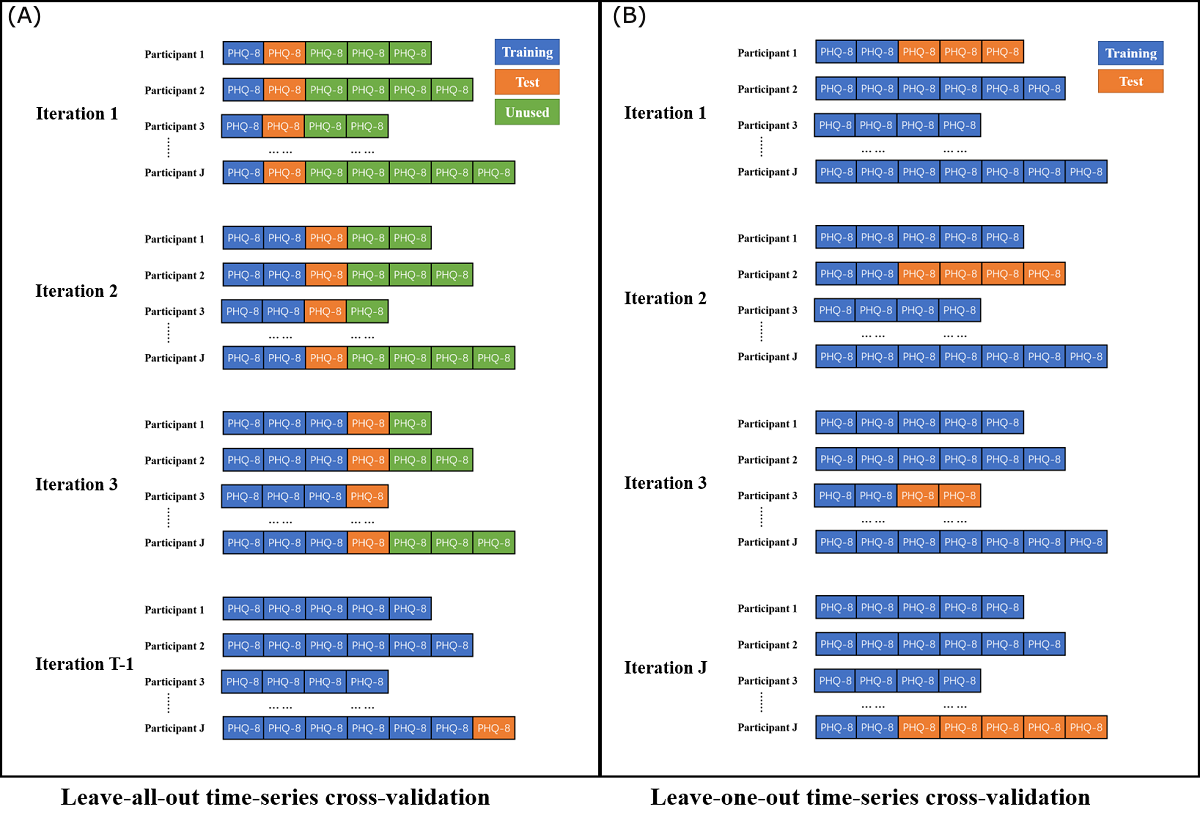
Two schematic diagrams of leave-all-out time-series cross-validation (A) and leave-one-out time-series cross-validation (B), where T is the maximum number of PHQ-8 intervals of one participant, J is the number of participants, the training set is indicated by blue, the test set is indicated by orange, and unused data are indicated by green. PHQ-8: 8-item Patient Health Questionnaire.

We selected root mean squared error (RMSE) and the predicted coefficient of determination (*R^2^*) as two metrics for model discrimination evaluation. As we used the temporal data, “future data” should not predict “past data.” Therefore, only the data observed before test data can be included in the training set. We applied leave-all-out (LAO) and leave-one-out (LOO) time-series cross-validation [48]. As the number of PHQ-8 intervals of each participant in our data was different, we made some minor modifications to these two schemes (Figure 5).

##### LAO Time-Series Cross-Validation

Each participant’s data were divided into a sequence of *t* consecutive same-sized test sets, where the size of each test set is the length of one PHQ-8 interval (14 days) and *t* is the number of PHQ-8 intervals of this participant. The corresponding training set included all PHQ-8 intervals before each test set. Then, test sets and training sets were pooled across all participants. This process generated *T*-1 test and training set pairs (no prior data to predict the first PHQ-8 score), where *T* is the maximum number of PHQ-8 intervals of one participant in our data set (*t*≤*T*).

##### LOO Time-Series Cross-Validation

Each participant’s data were divided into a training set and a test set. The training set was constructed using the first two PHQ-8 intervals of a participant, with the test set containing the rest of the participant’s PHQ-8 intervals. Then, the training set was pooled with all data from all other participants. This scheme generated *J* training and test set pairs, where *J* is the number of participants in our data set.

## Results

### Data Summary

**Figure 6:**
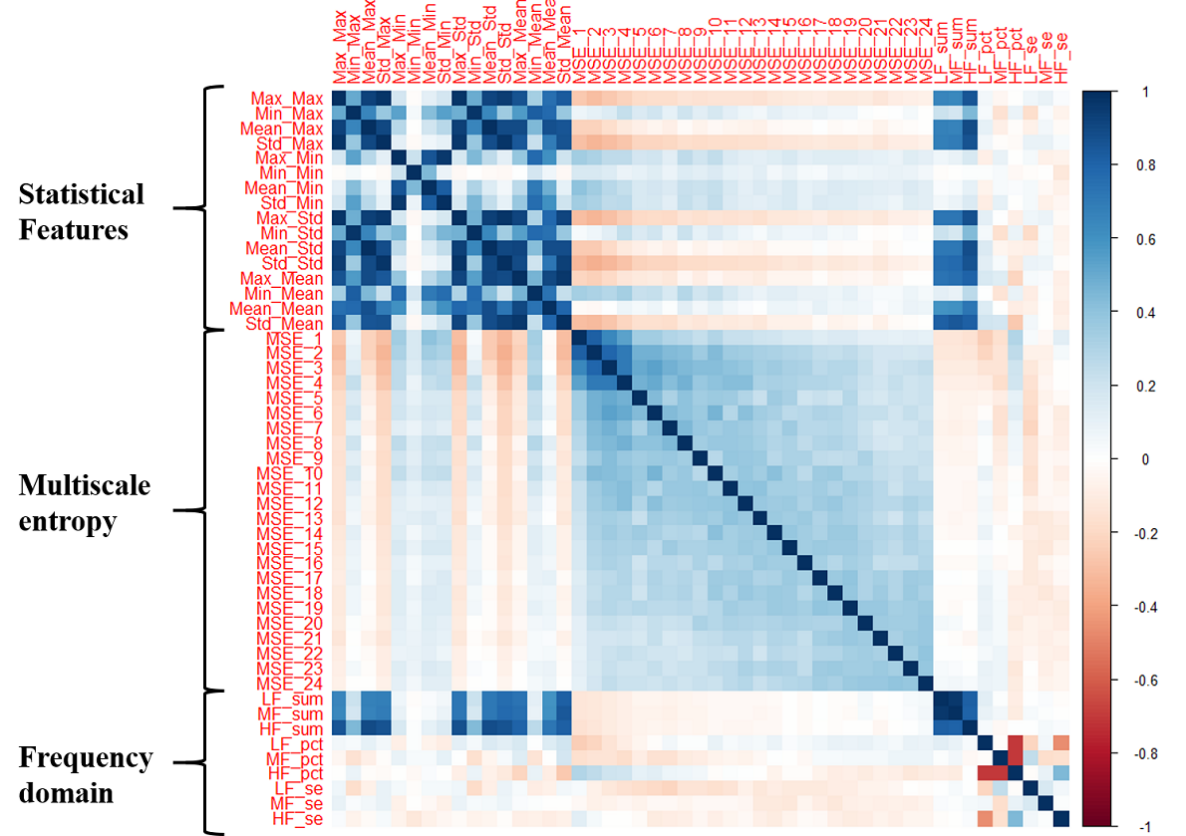
A correlation plot of pairwise Spearman correlations between all 49 Bluetooth features. Definitions of Bluetooth features in this figure are shown in Table 1.

**Figure 7:**
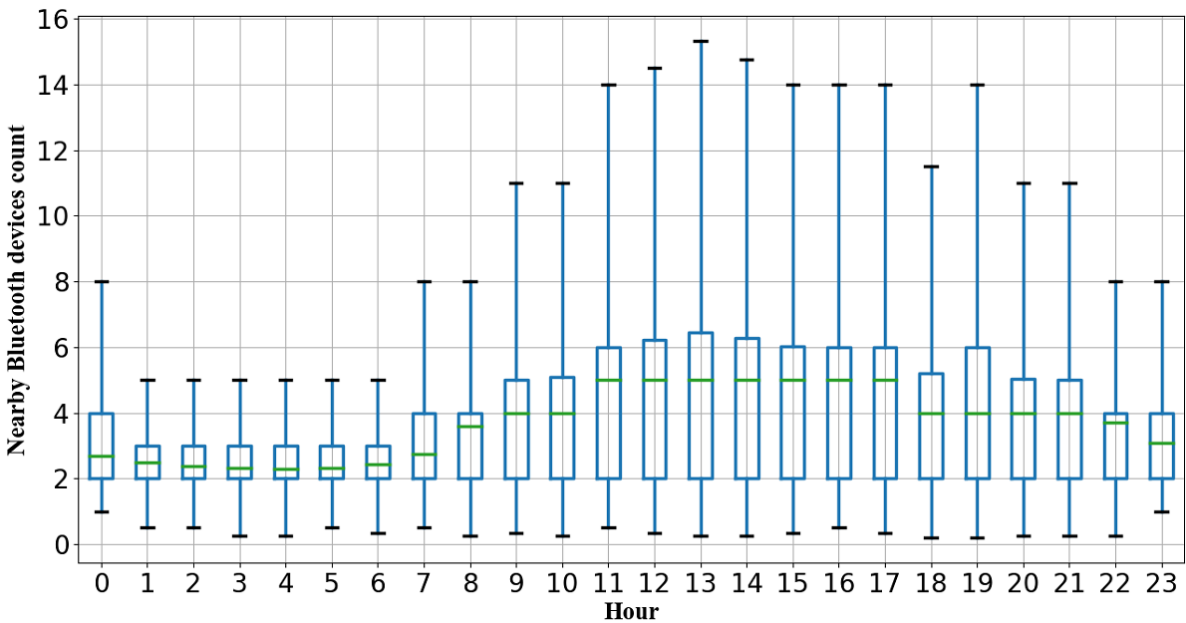
Boxplots of the nearby Bluetooth devices count (NBDC) for every hour in the whole population. Boxes extend between the 25th and 75th percentiles, and green solid lines inside the boxes are medians. Note the relative stationary NBDC during the night-time hours.

According to our date inclusion criteria, from June 2018 to February 2020, 2886 PHQ-8 intervals from 316 participants collected from three study sites were selected for our analysis. Table 2 shows the descriptive statistics for all 49 Bluetooth features, and Figure 6 presents pairwise Spearman correlation coefficients between all features. Table 3 presents a summary of the demographics and distribution of PHQ-8 records of all selected participants. Figure 7 presents boxplots of the NBDC for every hour in the whole population.

**Table 2:** Descriptive statistics for all 49 Bluetooth features.

Feature^a^		Mean	SD	Min	Q1	Median	Q3	Max
**Second-order statistics**								
	Max_Max	49.79	48.48	1.00	25.00	40.00	60.00	621.00
	Min_Max	5.09	6.22	0.00	2.00	4.00	6.00	90.00
	Mean_Max	18.56	18.94	0.75	9.23	14.07	21.62	268.29
	Std_Max	13.14	14.05	0.00	6.14	10.45	16.22	195.19
	Max_Min	1.59	2.08	0.00	0.00	1.00	2.00	43.00
	Min_Min	0.06	0.27	0.00	0.00	0.00	0.00	3.00
	Mean_Min	0.58	0.88	0.00	0.00	0.21	0.79	13.71
	Std_Min	0.50	0.62	0.00	0.00	0.42	0.70	11.94
	Max_Std	12.31	12.76	0.34	5.60	9.51	15.39	185.98
	Min_Std	1.20	1.45	0.00	0.56	0.87	1.32	21.61
	Mean_Std	4.55	4.87	0.16	2.17	3.25	5.24	70.65
	Std_Std	3.24	3.71	0.09	1.34	2.43	4.04	62.52
	Max_Mean	9.32	9.34	0.17	4.38	6.88	11.04	136.10
	Min_Mean	1.88	2.14	0.00	0.50	1.42	2.50	32.00
	Mean_Mean	4.42	4.19	0.07	2.19	3.40	5.28	49.55
	Std_Mean	2.13	2.59	0.05	0.84	1.45	2.54	49.37
**Multiscale entropy (MSE)**								
	MSE_1	0.80	0.46	0.05	0.42	0.71	1.13	2.44
	MSE_2	0.97	0.54	0.04	0.56	0.85	1.31	3.58
	MSE_3	1.12	0.66	0.09	0.70	1.01	1.42	9.41
	MSE_4	1.23	0.69	0.05	0.82	1.15	1.51	8.83
	MSE_5	1.35	0.82	0.10	0.93	1.27	1.62	8.51
	MSE_6	1.38	0.84	0.08	0.97	1.28	1.63	8.00
	MSE_7	1.47	0.97	0.10	1.01	1.33	1.70	7.72
	MSE_8	1.50	1.07	0.10	1.00	1.30	1.67	7.40
	MSE_9	1.58	1.22	0.10	0.99	1.32	1.72	7.30
	MSE_10	1.58	1.23	0.08	0.97	1.30	1.72	7.08
	MSE_11	1.58	1.29	0.09	0.95	1.25	1.67	7.02
	MSE_12	1.59	1.33	0.10	0.92	1.23	1.66	6.70
	MSE_13	1.74	1.46	0.11	0.98	1.30	1.79	6.55
	MSE_14	1.85	1.53	0.11	1.01	1.36	1.87	6.70
	MSE_15	1.96	1.62	0.13	1.03	1.39	1.95	6.55
	MSE_16	1.98	1.62	0.13	1.03	1.39	1.95	6.40
	MSE_17	2.04	1.67	0.14	1.02	1.39	2.08	6.14
	MSE_18	2.03	1.65	0.15	1.01	1.39	2.08	6.04
	MSE_19	2.09	1.69	0.17	1.01	1.39	2.08	6.04
	MSE_20	2.09	1.67	0.17	0.98	1.39	2.08	5.94
	MSE_21	2.10	1.66	0.18	0.98	1.39	2.20	5.83
	MSE_22	2.13	1.68	0.18	0.98	1.39	2.30	5.83
	MSE_23	2.17	1.69	0.18	0.98	1.39	4.28	5.61
	MSE_24	2.27	1.70	0.20	0.98	1.39	4.28	5.35
**Frequency domain (FD)**								
	LF^b^_sum	330.66	2469.74	0.05	17.41	53.87	184.80	85956.16
	MF^c^_sum	157.24	1166.32	0.02	8.16	25.77	83.05	34970.35
	HF^d^_sum	602.22	3272.44	0.47	55.72	151.74	403.38	64127.16
	LF_pct^e^	0.25	0.10	0.03	0.17	0.23	0.31	0.63
	MF_pct	0.13	0.10	0.01	0.07	0.11	0.17	0.74
	HF_pct	0.62	0.15	0.12	0.53	0.64	0.72	0.92
	LF_se^f^	0.83	0.10	0.38	0.78	0.85	0.90	1.00
	MF_se	0.82	0.09	0.40	0.77	0.83	0.88	0.99
	HF_se	0.90	0.04	0.72	0.88	0.90	0.92	0.99

^a^Definitions of Bluetooth features in this table are shown in Table 1.

^b^LF: low frequency (0-0.75 cycles/day).

^c^MF: middle frequency (0.75-1.25 cycles/day).

^d^HF: high frequency (>1.25 cycles/day).

^e^pct: percentage of spectrum power.

^f^se: spectral entropy.

**Table 3:** Summary of the demographics and 8-item Patient Health Questionnaire (PHQ-8) record distribution of all selected participants.

Characteristic		Value
Number of participants		316
**Demographics**		
	Age at baseline, median (Q1, Q3)	51.0 (35.0, 59.0)
	Female sex, n (%)	234 (74.1%)
	Number of years in education, median (Q1, Q3)	16.0 (14.0, 19.0)
**PHQ-8 record distribution**		
	Number of PHQ-8 intervals	2886
	Number of PHQ-8 intervals for each participant, median (Q1, Q3)	8.0 (3.0, 14.0)
	PHQ-8 score, median (Q1, Q3)	9.0 (5.0, 15.0)

### Association Analysis Results

The significant associations between depression severity (the PHQ-8 score) and Bluetooth features are presented in Table 4.

#### Associations Between the PHQ-8 Score and Second-Order Statistical Features

There were 10 second-order statistical features significantly associated with the PHQ-8 score. All these significant associations were negative, that is, the larger the value of these features, the lower the PHQ-8 score. Notably, *Min_Max* (the minimum value of daily maximum NBDC in the past 14 days) had the strongest association (z=−4.431, *P*<.001), which indicated that participants with a lower PHQ-8 score tended to have more daily social activities (such as social interactions and traveling) in the past 2 weeks. In addition, four features related to daily variance (*Max_Std*, *Min_Std*, *Mean_Std*, and *Std_Std*) of the NBDC were all significantly and negatively associated with depression.

#### Associations Between the PHQ-8 Score and Multiscale Entropy Features

MSE at scale 1, scale 2, and scale 3 (*MSE_1*, *MSE_2*, and *MSE_3*) were significantly and positively associated with the PHQ-8 score, while MSE at scale 16 and scale 22 (*MSE_16* and *MSE_22*) were significantly and negatively associated with depressive symptom severity. According to the explanations of MSE we mentioned in the Methods section, these associations indicated that participants with more irregular and chaotic NBDC sequences were likely to have more severe depressive symptoms, while those with periodic and regular NBDC sequences may have lower PHQ-8 scores.

#### Associations Between the PHQ-8 Score and FD Features

There were five FD features significantly associated with the PHQ-8 score. The spectrum power was related to both the amount and frequency components of the NBDC sequence, so it had relatively strong correlations with second-order statistical features (Figure 6). Therefore, the spectrum power of three frequency intervals (*LF_sum*, *MF_sum*, and *HF_sum*) were all significantly and negatively associated with the PHQ-8 score. Among them, the *MF_sum* had the strongest association (z=−4.766, *P*<.001) with depression, which indicated that the circadian rhythm of the NBDC sequence is important to reflect the severity of depression. Likewise, the percentage of middle-frequency power (*MF_pct*) was significantly and negatively associated with depressive symptom severity. The spectral entropy of HF (*HF_se*) was significantly and positively associated with depression. This indicated that participants with irregular short-term (<1 day) rhythms were likely to have more severe depressive symptoms.

**Table 4:** Coefficient estimates, standard error, z-test statistics, and *P* values from pairwise linear mixed-effect models for exploring associations between Bluetooth features and the depressive symptom severity (8-item Patient Health Questionnaire).

Feature^a^		Estimate	SE	z score	Adjusted *P* value^b,c^
**Second-order statistics**					
	Min_Max	−0.052	0.012	−4.431	<.001
	Mean_max	−0.016	0.006	−2.809	.005
	Max_Std	−0.015	0.006	−2.657	.008
	Min_Std	−0.215	0.056	−3.838	<.001
	Mean_Std	−0.065	0.023	−2.802	.005
	Std_Std	−0.048	0.020	−2.385	.02
	Max_Mean	−0.030	0.008	−3.498	<.001
	Min_Mean	−0.093	0.046	−2.036	.04
	Mean_Mean	−0.083	0.026	−3.225	.001
	Std_Mean	−0.095	0.027	−3.464	.001
**Multiscale entropy (MSE)**					
	MSE_1	0.642	0.225	2.853	.005
	MSE_2	0.433	0.192	2.255	.02
	MSE_3	0.401	0.202	1.985	.04
	MSE_16	−0.102	0.042	−2.429	.01
	MSE_22	−0.123	0.043	−2.860	.005
**Frequency domain (FD)**					
	LF^d^_sum	−0.021	0.005	−3.865	<.001
	MF^e^_sum	−0.067	0.014	−4.766	<.001
	HF^f^_sum	−0.027	0.010	−2.606	.009
	MF_pct^g^	−1.834	0.812	−2.259	.02
	HF_se^h^	3.821	1.820	2.099	.04

^a^Definitions of Bluetooth features in this table are shown in Table 1.

^b^Only significant associations (adjusted *P* value <.05) are reported.

^c^*P* values were adjusted by the Benjamini-Hochberg method for correction of multiple comparisons.

^d^LF: low frequency (0-0.75 cycles/day).

^e^MF: middle frequency (0.75-1.25 cycles/day).

^f^HF: high frequency (>1.25 cycles/day).

^g^pct: percentage of spectrum power.

^h^se: spectral entropy.

### Results of Likelihood Ratio Tests

The results of the likelihood ratio tests are presented in Table 5. Model B (with second-order statistical Bluetooth features) and model C (with all Bluetooth features) fitted data significantly better than model A (without Bluetooth features), indicating that Bluetooth features could improve the statistical model significantly. The goodness of fit of model C was significantly better than that of model B, indicating that nonlinear Bluetooth features (MSE and FD features) provided additional information to the statistical model.

**Table 5:** Results of the likelihood ratio tests of the three nested linear mixed-effect models.

Model	Difference of parameters	Chi-square^a^	*P* value
Model B^b^ vs model A^c^	16	31.04	.01
Model C^d^ vs model A	49	135.19	<.001
Model C vs model B	33	104.15	<.001

^a^The critical values of the likelihood ratio statistic are as follows: *χ^2^_0.05_*(16)=26.296, *χ^2^_0.05_*(33)=47.400, and *χ^2^_0.05_*(49)=66.339.

^b^Predictors of model B: demographics + 16 second-order statistical features.

^c^Predictors of model A: demographics.

^d^Predictors of model C: demographics + 16 second-order statistical features + 24 multiscale entropy features + nine frequency domain features.

### Performance of Prediction Models

A subset of 183 participants was selected for the prediction models. The results of the LAO and LOO time-series cross-validation are presented in Table 6. The *R^2^* score of the baseline model was 0.338 in LAO time-series cross-validation, which showed that more than 30% variance could be explained by the last observed PHQ-8 score and baseline demographics. In LOO time-series cross-validation, the *R^2^* score of the baseline model was negative, which indicated that the baseline model did not explain any variance in the LOO time-series cross-validation. To assess the improvement from nonlinear Bluetooth features, we tested the hierarchical Bayesian model with and without nonlinear Bluetooth features separately.

**Table 6:** Results of the leave-all-out time-series cross-validation and leave-one-out time-series cross-validation of the hierarchical Bayesian linear regression model, commonly used machine learning models, and the baseline model.

Model	Leave-all-out		Leave-one-out	
	*R^2^*	RMSE^a^	*R^2^*	RMSE
Baseline model^b^	0.338	4.547	−0.074	5.802
LASSO regression	0.458	4.114	0.144	5.178
XGBoost regression	0.464	4.092	0.346	4.523
Hierarchical Bayesian linear (second-order statistical features)	0.481	4.026	0.353	4.501
Hierarchical Bayesian linear (all Bluetooth features)	0.526	3.891	0.387	4.426

^a^RMSE: root mean squared error.

^b^The baseline model is the hierarchical Bayesian linear regression model with only the last observed 8-item Patient Health Questionnaire score and demographics as predictors.

In the subset, the maximum number of PHQ-8 intervals of one participant was 27, so the LAO time-series cross-validation went through *T*-1=26 iterations. The hierarchical Bayesian linear regression model with all Bluetooth features achieved the best result (*R^2^*=0.526, RMSE=3.891), beating the LASSO and XGBoost regression models. Compared with the result of the baseline model (*R^2^*=0.338), the improvement in the *R^2^* score was 0.188, which means the Bluetooth features explained an additional 18.8% of data variance. The nonlinear Bluetooth features explained an additional 4.5% of data variance in the hierarchical Bayesian model.

The number of subset participants was 183, so *J*=183 iterations of the LOO time-series cross-validation were performed. The hierarchical Bayesian linear model with all Bluetooth features had the best performance (*R^2^*=0.387, RMSE=4.426), but the result was close to that of the XGBoost regression model (*R^2^*=0.346, RMSE=4.523).

The performance of the hierarchical Bayesian linear regression model evaluated by the LAO cross-validation was better than the LOO cross-validation performance. One potential reason is that only the first two PHQ-8 intervals of one participant were used for training in the LOO cross-validation, which may have caused the model to underfit the patterns at the participant level.

## Discussion

### Principal Findings

This paper explored the value of the NBDC data in predicting depression severity. Compared with previous Bluetooth-related studies [15-18], our study was performed on a larger (N=316) multicenter data set with a longer follow-up (median 4 months). We extracted 49 features from the NBDC sequences in the following three categories: second-order statistical features, MSE features, and FD features. To the best of our knowledge, this is the first time that MSE and FD features have been used in NBDC and depression data analyses. According to the results of association analyses (Table 4), when depression symptoms worsened (increase in the PHQ-8 score), one or more of the following changes were seen in the preceding 14 days of the NBDC sequence: (1) the amount decreased, which is consistent with the finding by Wang et al [15], (2) the variance decreased, (3) the periodicity (especially the circadian rhythm) decreased, and (4) the NBDC sequence became more irregular and chaotic.

These changes in the NBDC data can be explained by depression symptoms. The main manifestations of depression include negative feelings (such as sadness, guilt, stress, and tiredness) and loss of interest or pleasure [49]. This may lead to changes in behaviors, such as increased time at home [29,50], decreased mobility [3,29], loss of the ability to work or study [2,49], reduced intensity of social interactions [1], unstable and irregular sleep [51], and decreased engagement in activities [52]. The increased time at home, inability to work or study, and diminished social interactions are reflected in the reduced amount of the NBDC sequence. The decreased mobility and engagement in activities may be possible reasons why participants with higher PHQ-8 scores have lower variance-related features (*Max_Std*, *Min_Std*, *Mean_Std*, and *Std_Std*). Depression also may lead to misalignment of the circadian rhythm and make people’s life rhythms (such as sleep rhythms and social rhythms) more irregular [19]. This can be reflected in reduced periodicity and increased irregularity of the NBDC sequence. Saeb et al [29] and Farhan et al [30] found similar findings in GPS data, and showed that the circadian rhythm of the GPS signal was significantly and negatively correlated with depression.

From the perspective of the statistical model, Bluetooth features extracted in this paper significantly improved the goodness of fit for the PHQ-8 score, and nonlinear Bluetooth features (MSE and FD features) can provide additional information to second-order statistical features (Table 5). From the perspective of the prediction model, these 49 Bluetooth features explained an extra 18.8% of the variance in the PHQ-8 score relative to the baseline model, containing only the last PHQ-8 score and demographics, and MSE and FD features explained an extra 4.5% of data variance in the hierarchical Bayesian model (Table 6). From the perspective of the correlations between Bluetooth features (Figure 6), we can observe that, except for three FD features related to the spectrum power that had relatively strong correlations with second-order statistical features, the correlations between other nonlinear Bluetooth features and second-order statistical features were not obvious. This indicated that the MSE and FD features captured dimensions of information to second-order statistical features.

In our prediction model, the hierarchical Bayesian linear regression model achieved the best results in both the LAO and LOO time-series cross-validation. Compared with other models, one of the advantages of the hierarchical Bayesian model is that it performs individual predictions while considering the population’s common characteristics [43]. Therefore, the hierarchical Bayesian model can be considered a suitable prediction modelling method for longitudinal data. The LOO time-series cross-validation results illustrated that the hierarchical Bayesian model could predict depression for participants with few observations (only two PHQ intervals in the training set) that overcomes the cold start problem. The hierarchical Bayesian linear model achieved a better result in the LAO time-series cross-validation, which indicated that the prediction results gradually became more accurate and individualized when each participant had more data available in the training set.

### Limitations

The RADAR-MDD project was designed for long-term monitoring (up to 2 years) and collecting many other passive data, such as GPS data, acceleration data, app usage, and screen lightness, which need to be collected simultaneously through the mobile phone. Therefore, to avoid excessive battery consumption, nearby Bluetooth devices were scanned hourly in this study. However, some past studies suggested scanning nearby Bluetooth devices every 5 minutes to achieve high enough temporal resolution [9,18]. Although hourly NBDC data can also reflect individuals’ behaviors and statuses, our lower data resolution may cause the loss of some dynamic information. On the other hand, using the relatively low resolution enabled us to collect multimodal data without excessive battery consumption. As the NBDC data are related to individuals’ movement and location information, we will combine the NBDC data with GPS and acceleration data for future analysis to understand the context of the Bluetooth data.

As we mentioned in the Methods section, the MAC addresses and types of Bluetooth devices were not recorded for private issues. This made it impossible to distinguish between mobile phones and other Bluetooth devices (such as headphones, printers, and laptops), and between strangers’ and acquaintances’ devices. The advantage of the NBDC data is that the data contain mixed and rich information. The disadvantage is that it is difficult to explain the specific reasons for changes in the NBDC, that is, we cannot know whether the changes in the NBDC are caused by social interactions, working status, traveling, or isolation. Therefore, this paper did not explain in depth the actual meaning behind the Bluetooth features. For this limitation, we plan to use hashed MAC addresses in future research.

For the FD features, the division of the frequency intervals of the spectrum of the NBDC sequence in this paper was manually specified by our experience. The purpose of extracting these FD features was to prove that the NBDC sequence’s FD has the potential to provide more information about individuals’ behaviors and life rhythms. It is necessary to discuss the optimal boundaries of frequency intervals of the NBDC data in future research.

This paper applied the hierarchical Bayesian linear regression model to explore the linear relationships between Bluetooth features and depression. However, there may be nonlinear relationships between social connections and depressive symptom severity. The Gaussian process [53], using the kernel method to find nonlinear relationships, will be considered in future research.

### Conclusion

Our statistical results indicated that the NBDC data have the potential to reflect changes in individuals’ behaviors and statuses during a depressive state. The prediction results demonstrated that the NBDC data have significant value in predicting depressive symptom severity. The nonlinear Bluetooth features proposed in this paper provide additional information to statistical and prediction models. The hierarchical Bayesian model is an appropriate prediction model for predicting depression with longitudinal data, as both participant-level and population-level characteristics are considered in the model. These findings may support the mental health monitoring practice in real-world settings.

## Abbreviations

CIBER: Centro de Investigación Biomédican en Red
FD: frequency domain
HF: high frequency
KCL: King’s College London
LAO: leave-all-out
LF: low frequency
LOO: leave-one-out
MAC: Media Access Control
MF: middle frequency
MSE: multiscale entropy
NBDC: nearby Bluetooth device count
PHQ-8: 8-item Patient Health Questionnaire
RADAR-CNS: Remote Assessment of Disease and Relapse - Central Nervous System
RADAR-MDD: Remote Assessment of Disease and Relapse - Major Depressive Disorder
RMSE: root mean squared error
RMT: remote measurement technology
VUmc: Vrije Universiteit Medisch Centrum
